# Advances and Applications of Block Copolymers

**DOI:** 10.3390/polym15132930

**Published:** 2023-07-02

**Authors:** Nikolaos Politakos, Apostolos Avgeropoulos

**Affiliations:** 1 POLYMAT, Applied Chemistry Department, Faculty of Chemistry, University of the Basque Country, UPV/EHU, Paseo Manuel de Lardizabal 3, 20018 Donostia-San Sebastián, Spain; 2 Department of Materials Science Engineering, University Campus-Dourouti, University of Ioannina, 45110 Ioannina, Greece; aavger@uoi.gr; 3 Faculty of Chemistry, Lomonosov Moscow State University (MSU), GSP-1, 1-3 Leninskiye Gory, 119991 Moscow, Russia

Polymers are materials that have constantly evolved from the beginning of their discovery until the present day. During the last few decades, polymers have been used in many areas of science and cutting-edge research and have been applied to everyday items. Many polymerization techniques, either synthetic or natural, exist to create molecularly homogeneous materials with specific properties prepared from different types of organic or even inorganic monomers.

The most important class of polymers is block copolymers (BCPs) since they can be prepared through different methods and exhibit various properties combined with their chemically different segments. The importance of block copolymers can be supported by the multitude of related manuscripts published on a daily basis, their use in practical applications in almost all areas, and novel discoveries based on BCPs in many aspects of life. Block copolymers can be used as composites and hybrid materials, providing new properties and, in some cases, responsiveness in terms of temperature, humidity, pH, redox, magnetic, electric, swelling, light, and others. Block copolymers are also very important for self-assembly studies since they can be organized into nanostructures and may be used in applications such as lithography or membranes. In general, BCPs have been used in medicine, chemistry, nanotechnology, physics, water treatment, environmental applications, wound healing, solar cells, photocatalysis, and many other fields.

Nevertheless, block copolymers face new challenges and problems every day. This Special Issue brings together different experimental works and reviews and attempts to cover the majority of the recent advances and applications of block copolymers in the last few years.

This Special Issue has gathered scientific works from research groups examining various copolymers, indicating advances in architecture, functionalities, and applications. The total number of manuscripts (17) published in this Special Issue indicates the importance of BCPs and the fact that many research groups and relevant members of the scientific community are thoroughly interested in the advancement of block copolymers and their applications.

In one study, copolymers with PVP (poly(vinylpyridine)) and its partially quaternized derivatives were studied based on the structure/properties relationship, focusing on solvation and optical properties [1].

Research has also been carried out on fabricating well-ordered epoxy resin nanonetworks modified with poly(butyl acrylate)-b-poly(methyl methacrylate) (PBA-b-PMMA) to enhance energy dissipation by exploiting the self-assembly of polystyrene-b-poly(dimethylsiloxane) (PS-b-PDMS) with gyroid and diamond structures as templates. Selectively etching a PDMS block can provide nanochannels for the polymerization of epoxy resin. A PBA-b-PMMA BCP can be self-assembled into a spherical nanosized micelle in the epoxy matrix, which acts as a toughening agent for forming soft domains [2].

A process of combining anionic polymerization with ROP copolymers based on PLA was carried out by a group of researchers, providing new insights into polymer design strategies for high-performance PLA-based materials using stereo complexation [3].

In one study, copolymers of PBA-b-PDMAEMA (butyl methacrylate as a hydrophobic monomer and 2-(Dimethylamino) ethyl methacrylate) were prepared by surface-initiated ATRP from SiO_2_ and used as a hybrid adsorbent for heavy metals (Cr) and phenol [4].

In another study, linear and star block copolymer nanoparticles of (polystyrene-b-poly(4-vinyl pyridine))n (PS-b-P4VP)n were prepared via RAFT, and their nano-assemblies in toluene were studied. Polymerization kinetics were investigated, and the effect of the arm number of the PS/P4VP chain on the block copolymer nano-assemblies prepared via PISA was explored by changing the length of the PS or the P4VP [5].

Two manuscripts focusing on crystallization are also included in this Special Issue. The first study [6] investigates the crystallization of the α and γ forms of iPP in propene–butene copolymers by analyzing the thermodynamics and kinetics effects, whereas the second study [7] is dedicated to the crystallization behavior of isotactic propene–octene copolymers (iPPC8) synthesized with a metallocene catalyst. The effect of the octene units excluded from the crystals on the crystallization of the α and γ forms of iPP was analyzed and compared to those of the different aforementioned comonomers that are partially included in the crystals of the α and γ forms with different degrees of incorporation.

The synthesis of hybrid materials based on AA grafted in aqueous collagen dispersions was also investigated in one study [8], whereby the determination of the molecular weight characteristics, structure, mechanical properties, and cytotoxicity of the obtained copolymers was carried out, and the authors evaluated the prospects for using the obtained copolymers in scaffold technologies.

Block copolymers can also be a powerful new precursor for fabricating porous carbon structures (PCFs) for electrochemical performance. Different copolymers can provide different nanostructures and thus improve electrochemical performance. Based on the above, a study focusing on the copolymer PAN−b−PS and carbon powder was presented as a comparative study of two different electrode materials for supercapacitors [9].

Additionally, the formation of photo-functional nanoparticles (owing to the intrinsic properties of the porphyrin) through the electrostatic complexation between the poly(2-(dimethylamino) ethyl methacrylate)-b-poly[(oligo ethylene glycol) methyl ether methacrylate] (PDMAEMA-b-POEGMA) double hydrophilic block copolymer or its quaternized strong polyelectrolyte counterpart (QPDMAEMA-b-POEGMA) was investigated [10]. This study examined their solution properties by changing the porphyrin content, photophysical characteristics, and photosensitivity under different temperatures and pHs.

Moreover, a study on mixtures consisting of conjugated polymers and reduced graphene oxide was also published with the major aim of tuning the energy gaps and revolutionizing various electronic applications, such as organic solar cells and OLED displays [11]. Another study is also presented to shed light on the temperature dependence of the Flory–Huggins interaction parameter χ(T) between the two acrylic monomer species of BA and MMA by small-angle X-ray scattering (SAXS). Well-defined copolymers were prepared via ATRP, and the study’s authors measured the order–disorder transition (ODT) temperature within the investigated temperature range [12]. In terms of theory and simulations, the phase behaviors of molten A–b–B copolymers in the weak segregation regime were also investigated using equivalent free energies. In this study, the theoretical calculations were compared with the experimental results [13].

Furthermore, the self-assembly behavior of different terpolymers with the PB1,2 segment modified was studied within the scope of chemical modifications. The as-synthesized polymer brushes from the polydiene segment can have possible applications in nanotechnology [14].

In addition, in another study, the nanostructure of a bio-based epoxy thermosetting formulation was investigated by using epoxy resin modified using different amounts of the PEO–PPO–PEO triblock copolymer. The effect of the copolymer amount on the morphology of the nanostructured thermosetting system was analyzed through atomic force microscopy (AFM) [15].

Finally, two reviews are presented in this Special Issue regarding block copolymers. The first review investigates the regulation of block copolymer self-assembly structures by exploring the factors that affect the self-assembly nanostructure. This review highlights block-copolymer-based mass transport membranes, which play the role of “energy enhancers” in concentration cells, fuel cells, and rechargeable batteries [16].

The second review explores block copolymers’ significant contribution to 3D printing in recent years by focusing on bio-applications. It is shown in the study that block copolymers constantly evolve through new synthetic approaches and through the introduction of new blocks with stimulating properties. This review aims to manifest the use of block copolymers as a leading evolving player in 3D/4D printing and show the significant contribution of block copolymers in bio-applications with specific advances [17].

Therefore, it may be concluded that block copolymers play a significant role in the advancement of research in polymer science and engineering. This Special Issue focuses on various matters from different areas of block copolymer research, showing advances and applications in self-assembly, crystallization, synthesis, chemical modification, 3D printing, membranes, energy materials, nanotechnology, hybrid materials, micelles, order–disorder transition, and biomaterials. As guest editors of this Special Issue, we are confident that a significant contribution to the field of block copolymers will be made through this collection of studies.
